# On Cholera

**Published:** 1866-09

**Authors:** A. Clark

**Affiliations:** Professor of Pathology and Practical Medicine, College of Physicians and Surgeons, N.Y.


### ON CHOLERA.

By A. CLARK, M.D., Professor of Pathology and Practical Medicine, College of Physicians and Surgeons, N.Y.

### THE TREATMENT OF CHOLERA.

We may now inquire whether any treatment is curative in the several stages of cholera. It is generally believed that the diarrhœa of the first stage, if sufficiently prolonged to permit the application of medical precepts, is as easily cured as a diarrhœa of any other kind. This is not quite true. A diarrhœa in cholera seasons is more dangerous and less uniformly manageable than in ordinary seasons when cholera is not prevailing. At the same time, here is the great field of professional usefulness; this is the period of the disease when cures should be effected. Then what are you to do? First, send the patient to bed, and let him have the advantage of warmth and rest in the recumbent position. It is well to obtain a little diaphoresis —not profuse sweating, but a soft skin, or gentle perspiration, such as may be caused by a warm bath, or better, by a foot bath. The administration of cathartics is not generally thought proper; and this is especially true of the salines. Indeed all cathartics, while cholera is threatening, should be avoided as far as possible in those not attacked. Many an instance has come to my knowledge of cholera apparently precipitated by the administration of medicines of this class. Castor-oil is the least objectionable, and I can conceive may, under certain circumstances, be serviceable; for example, when a diarrhœa has been provoked by accumulated fæces in the intestines, or by errors of diet, or by the indigestable portions of certain fruits. In general, the first aim is to check the diarrhœa. Among the medicines that we use for this purpose, opium or an opiate almost always finds a place. A favorite prescription in past epidemics is the following:—"Tinct. opii, spt. camphor, tinct. capsici, tinct. catechu, tinct. caryoph., equal parts. Of this a teaspoonful more or less frequently according to the urgency of the symptoms.* Those who entertain the view that this

*Dr. Squibb, in "Advice upon Epidemic Cholera," lately published, gives the following formula, which he thinks more useful by the addition of chloroform. He proposes to call the preparation the "Compound Tincture of Opium" or is a disease which replaces miasmatic fevers, advocate the use of quinine. Indeed, it is claimed that quinine alone will suppress the discharges. The astringent preparations of iron have some reputation, as the peritrate, or the persulphate, with or without quinine. A pill that Dr. Houston thinks as serviceable as any, is made up thus:—Acetate of lead and camphor, each twenty-four grains; acetate of morphia, two or three grains; oil of cinnamon, five drops; mucilage of gum arabic, sufficient. This is made into twelve pills, and one to be given every two, three, or four hours, according to the nature of the case. Another favorite prescription is what is called Hope's mixture. Its composition is as follows:—Nitric acid, one drachm; camphor mixture, eight ounces; these to be mixed, and afterwards the tincture of opium added to the amount of two scruples. One quarter of this makes a dose, to be repeated more or less frequently. If the diarrhœa begins after a surfeit, or there is reason to suppose there is undigested food in the stomach, a quick emetic is regarded as important. Alum, the aromatic confection, the compound spirits of ammonia, chalk mixture, hæmatoxylon, kino, and calomel in grain doses, are in the list of remedies for this stage; each, however, is given with opium or its tincture. But none of these medicines, regarded as astringents, are as highly valued by the English physicians as the acetate of lead. It is given with opium, one to eight grains of the acetate, and one-quarter of a grain to a grain of opium; in the small doses every hour. Some physicians claim success in the use of the pulvis cretæ compositus cum opio; others prefer hydrargyrum cum creta, and Dover's powder. These will, some of them probably, answer your purpose; but rest in bed is regarded as more important, perhaps than any special medication.

Passing to the serous diarrhœa—the symptom soon attended by cramps and vomiting—the question comes whether any particular remedy is specially serviceable. Dr. Fuller—the same, I believe, who has made a high reputation by his treatise on rheumatism—holds that sulphuric acid, first suggested by Mr. W. S. Cox, of Kensel Town, England, has almost sovereign power over this stage of the disease. His mode is to add to eleven ounces of water one ounce of dilute sulphuric acid, and to

"Diarrhœa Mixture." It is tincture of opium, spirits of camphor, and tincture of capsicum, each one fluid ounce; purified chloroform, three fluid drachms, and a sufficient quantity of stronger alcohol to make the whole measure five fluid ounces. He advises a teaspoonful as a dose, to be taken after each stool. If the diarrhœa increases he would double the dose. The mixture is suggested for popular use till a physician can be obtained.

give of this three tablespoons every half-hour; and he claims that it is infallible; but we hear of so many infallible remedies for cholera in its various stages that we have come to doubt infallibility, even in Dr. Fuller. With this, he sometimes combines in the alternate doses, half a drachm to a drachm of chloric ether. He occasionally commences the treatment by administering two grains of opium. He sometimes adds five grains of calomel in one dose. He also employs sinapisms to the stomach, and friction to the extremities. This treatment has the support of some good names in England. M. Worms, of Gros-Gaillou Hospital, uses sulphuric acid in sweetened water, one part to three or four or five hundred, as a drink, and attaches great importance to it. Dr. Hutchinson, however, in 1854, says:—"Sulphuric acid, so highly recommended by Dr. Fuller, was prescribed, but without any benefit."

Sulphur in substance has been proposed, and the suggestion comes from the idea entertained by some persons that the disease is caused by vegetable fungi. Dr. Grove takes of precipitated sulphur, one drachm; bicarbonate of soda, one drachm, compound spirits of lavender, six drachms, and of water two ounces, to make a mixture; of this he gives a tablespoonful every half or quarter of an hour. This is for him a most serviceable remedy. He says that it checks the serous discharges and restores warmth. He refers to the experience of some of the Scotch people, who treated themselves for what was supposed to be cholera with great success, using only brimstone and whiskey. He refers also to the *London Practice of Physic*, 1692, in which sulphur and the alcoholics are spoken of as the only means of curing a severe and fatal diarrhœa which prevailed in England about that time. The suggestion of Dr. Grove has the support of Dr. Cormack, and may be tried; but I fear it will share the fate of all cholera specifics.

Creasote, says Dr. Cormack, if the patient does not refuse to take it on account of its odor, hardly ever fails to arrest the serous discharges. Two or three drops given every hour or two, it is claimed, will constrict the vessels and stop the diarrhœa. This may be the language of hope, but it is not the language of science. That creasote may be serviceable is very possible; but to say it hardly ever fails, is but to bring discredit on him who says it.

In the treatment of the third stage of cholera, chloroform has gained considerable reputation. It was used by the late Professor Horner, of Philadelphia, in the cholera of 1849, and used with some success. Dr. Hartshorn, of Philadelphia, has presented to the profession what he regards as a useful formula:

℞. Chloroform,------------------------- f ℨ ij.

Spt. Camph.,------------------------

Tinct. opii, āā,------------------------ f ℨ iss.

Ol. cinnamon,---------------------- gtt. viij.

Alcohol,------------------------------ ℨ iii. M.

Professor Horner and Dr. Hartshorn gave chloroform in collapse, a few drops every few minutes, combined with camphor and laudanum, and the latter thinks its success extraordinary. Of Dr. Hartshorn's prescription five to thirty minims or more are to be given, with ice internally and warm applications externally. Dr. Davies (Dr. Gull's Report) had at one time very great confidence in chloroform in the stages of serous diarrhœa and in collapse, and gave it in doses of seven to ten drops every quarter or half-hour, claiming very great success; in nine cases of cholera and thirteen of the worst diarrhœa, only one death; in fourteen cases treated by Mr. Tower, under Dr. Davies' observation, only one death, and so on. In subsequent reports his trust in chloroform is gradually more and more shaken, till in his fourth and last he says he has lost confidence in it when used alone. On the whole, summing up all the reported experience which I have been able to find, it cannot be claimed that this medicine has any great curative power. It often allays the vomiting and cramps, and is worthy of further trial. Chloroform has been used by inhalation, and the effect of its use in that manner is to allay the cramps and suspend the vomiting, but unfortunately the patient must be kept continually in a state of slight anæsthesia, or these symptoms will return. Its administration does not seem to have materially diminished the mortality from cholera, though it has doubtless rendered the disease less painful, and may be worthy of use for that purpose.

Strychnine is very warmly advocated by Professor Fraser, of McGill College, Montreal, to be used in impending collapse, together with the means which tend to stop the discharges. He reports twenty-five cases treated by it, in doses of $\frac{1}{48}$ of a grain, dissolved in acetic acid and alcohol, repeated every quarter of an hour, sometimes every five minutes, until six or twelve doses are given, or till there is a reaction or specific effects. Twelve doses would make one-fourth of a grain. He thinks it has great power in sustaining the action of the heart and capillary vessels. His success he reports as twenty saved; and of the five others, one only died in collapse, the others in the stage of reaction. I have had no experience with the drug given as here suggested; but as it was used together with means to stop the discharges, and these means are not specified, it is pertinent to inquire how the credit of these cures should be divided. The suggestion was made near the end of the last epidemic (1854), and there has been little opportunity to test its value.

Now, of collapse. *Electricity* has been tried. It seems to revive the energies for a little time. The current has been passed from the neck to the epigastrium, to excite the action of the diaphragm, and from side to side to stimulate the heart. In one patient of Dr. Peacock, a child, the temperature in the mouth, rose from 88° to 92°, and the pulse became fuller under the influence of this agent, and the patient recovered; but the same physician was not able to sustain this success. Galvanism has a similar power. But, all considering, we may say of electricity and galvanism, that while in collapse they have the power of increasing warmth and bringing back the pulse temporarily, it is doubtful whether they have yet saved a single life. The *wet sheet*, used after the manner of the hydropathists, was tried pretty extensively in England during the epidemic of 1849. The result was a slight reaction, but it did not gain the grade of a curative agent. *Affusions* have been tried. Patients have been placed in a warm hip-bath, and both warm and cold water suddenly poured over the head, shoulders, and body. This is done quickly, and the patients are immediately placed in bed, in warm blankets. This plan has the power of producing some reaction, and is on the whole thought favorably of. Its effects are said to be better than those of the hot bath. *Oxygen* has been used in collapse by inhalation in a few instances, but it does not seem to have gained favor, because the temporary good effects have been followed by unfavorable results. The patients have almost all died. *Counter-irritation.*—Caspar dipped clothes in alcohol, laid them upon the abdomen, then set them on fire; but the patients did not get well for all that. Indeed it is striking that this sort of cauterization did not seem to produce much redness upon the surface to which it was applied. Milder counter-irritation, however, is generally resorted to; stimulating applications and warmth in various ways; mustard to the abdomen, the legs, and chest, particularly to the epigastrium, is hardly ever omitted. I do not suppose we can say that rubefacients, so used, have been of any *signal* service. They have the negative merit of innocence, and probably the positive one of arousing, in some degree, the failing nerve powers, and of quickening somewhat the capillary circulation. *External warmth*, notwithstanding it is often disagreeable to the patient—notwithstanding his cold arms feel heated—is pretty generally received with favor. A moderate degree of artificial heat aids in restoring to the body its natural temperature, and improves the chances of recovery. *Emetics* have been proposed—of common salt, or mustard, or sulphate of zinc, or ipicac.; and the result of experience seems to be that they do to a certain extent arouse the heart to activity, that they bring a little warmth to the surface, and for a moment distribute the blood a little more evenly through the body. But they are not curative; their effect is temporary, and the discomfort they produce is often considerable. They may be useful in another way. One who has seen little of the disease can hardly understand why the vomiting it produces should not prohibit the use of emetic drugs; but that vomiting, though it may cause the ejection of a great deal of fluid secretion from the stomach, is not forcible and violent, and often leaves undigested food in the organ. This is demonstrated in examinations after death. Such undigested food has been found after two days and more of vomiting. To remove this is to remove a source of irritation, and an emetic will often do it. *Ice, and cold drinks.*—A distinguished writer on the treatment of cholera remarks that it is gratifying to find that the experience of the last epidemic has confirmed the whole profession in the use of ice and cold drinks for those in collapse and approaching collapse. The burning thirst is allayed by swallowing portions of ice. It is true that we have to contend with the disposition to vomit; but the chances of the patient seem to be improved in two ways—by relieving suffering, and supplying to some extent water, which his blood lacks. Cold drinks and ice, then, have been received with extraordinary favor; and though not heroic measures, if they palliate only, they give nature a better opportunity to do a kind act. Dr. Murray, in India, states that *injections into the rectum, at a temperature of* 120°, were in his hands of great service in restoring warmth to the body and giving force to the pulse. He used a weak solution of common salt and carbonate of soda, a pint at a time, and repeated it every half-hour, or less frequently. The injections were usually retained but a few minutes, yet sufficiently long to produce recognizable effects. It may seem that giving cold fluids by the mouth, and heated ones by the rectum, cannot both be defended; that one would neutralize the effects of the other. But cold applied to the mucous membrane, in the upper part of the alimentary canal, diminishes the hyperæmia, the irritation, and the vomiting; while the injection falls upon a membrane less likely to be engorged, and probably increases the warmth of the body without doing any local mischief. Dr. Joel Foster, of this city, informs me that he has used warm injections for this purpose with advantage, and thinks he can cause them to be retained for a longer time by holding a compress against the anus.

Considerable success has been claimed in behalf of a *saline solution*, first proposed by Dr. Stevens, then of Jamaica. Believing that analysis showed some loss of saline constituents of the blood, it occurred to him that these could be restored by administering salts dissolved in water whenever the patient could retain liquids. Drs. Gull and Baly inform us that at the Cold-Bath Fields Prison other modes of treatment were tried, and the patients all died; the number was not very great, however, treated by other plans; for after five or six had died, the physicians adopted Dr. Stevens' method. The result was, that of patients so treated in the prison hospital, and in a lane in the neighborhood, forty-four out of forty-six recovered. This would be extraordinary success if the patients were all in the dangerous stage of the disease. Figures, as I have said, are uncertain guides when not attended by full explanations; yet we have but little else to trust, and so must do the best we can with them. The mixture used on this occasion was, bicarbonate of soda, half a drachm; common salt, one scruple; chlorate of potassa, seven grains; repeated every hour in such quantity of water as might seem expedient at the time of prescribing. Another solution has been used also, which is: common salt, half a drachm; tartrate of soda, twelve grains; phosphate of soda, eight grains; dissolved and taken at a draught. With reference to the appropriateness of these prescriptions, I may say a word when I come to another branch of the subject. This saline treatment of Stevens, though received with extraordinary favor in certain places, has not been successful in others, and the summing up by Gull and Baly is substantially this, that while at times it seems to have aided recovery materially, at other times it seems rather to have increased the evacuations and the danger. It has at least this merit—that when it is mainly relied upon, more dangerous medication is avoided. *Camphor* is a medicine that has been used pretty extensively in all the stages of this disease. In the collapse it has the reputation of allaying the cramps in a certain degree, and lessening the irritability of the stomach, and consequently the vomiting. It is certainly a safe medicine, and is believed to be as good a diffusible stimulant as can be used; and given in solution in chloroform, is perhaps of some advantage.

I have here a manuscript paper which was sent me in 1854 or 1855, by Dr. O. H. Smith, then of Williamsburgh, now of New York, giving a summary of the treatment of cholera in the hospital of which he had charge in 1854. It contains one statement which I think is worthy of consideration. I present it to you in his own words:—"Sulphuric acid, so highly eulogized by Dr. Fuller, of St. George's Hospital, was prescribed, but I could get no good results from its administration. Strychnine, as recommended by Prof. Fraser, of McGill College, was given, and I think it may have had some effect in postponing the period of collapse; but I do not think it had any control over the rice-water evacuations. I gave this remedy in view of the opinion that cholera poison acts especially on the ganglionic nerve centres of the abdomen; but its effects were such that I put it down as a doubtful as well as dangerous remedy. I tried the oxide of bismuth. It seemed to have no control over the watery discharges; but I think it relieved to some extent the retching and vomiting, so constant after the discharges had been arrested. In all cases the abdomen was enveloped in strong mustard poultices." Dissatisfied with all these agents and means, except the last, Dr. Smith turned to brandy.* "To restrain the rice-water evacuations," he says, "I have ordered an injection into the bowel, containing an ounce of brandy, two or three ounces of strong tea, and five or ten grains of the sugar of lead, to be repeated after every discharge. At the same time I ordered a soft pill, containing quinine, calomel, and camphor, of each half a grain, to be given every fifteen or twenty minutes; the whole abdomen being covered with mustard poultices. The patient was also directed to take brandy and water every five minutes by the mouth." The vomiting and dejections soon ceased. The feature of this treatment which has been but little if at all tried, is the injection of diluted brandy into the intestines, and it is to this that Dr. Smith attaches chief importance; but he found that brandy diluted with water was equally beneficial. His results induce me to commend his

* I have to-day (July 18) received a letter from Dr. Smith, in which he informs me that Drs. Walser and Bissel, in the quarantine ships, speak favorably of brandy injections; that Dr. Otis and McLean have each treated a case on his plan with good results. These cases will be published in the *Record*. Dr. Dalton, Sanitary Superintendent, informs me that as far as he can judge from three or four cases reported to him, there is a probability that these injections will be useful.

method to further trial, notwithstanding the general opinion which is adverse to the use of stimulants of this kind during the particular stage of which we are now treating. Dr. Smith here reports the cases of two children having rice-water discharges, to whom the parents administered two or three tablespoonfuls of brandy in each case, and made the children fairly "drunk;" the discharges ceased—none occurred after the full effects of the brandy were felt. These cases taken alone, would argue strongly in favor of brandy and the free use of it. But two cases will hardly affect a rule derived from the observation of several epidemics.

There is another mode of treatment, if it can be so called. A considerable number of physicians having charge of cholera hospitals, observing the ill-success of all plans of treatment, have felt justified in leaving a number of patients without any treatment besides cold drinks and ice to satisfy their thirst, and proper bedclothes. Dr. Hutchinson says, referring to some he left in this way, that "The most valuable experience derived from the observation of the recent epidemic (that of 1854) is, that cholera patients should be disturbed by remedies as little as possible. And when in any case we are at a loss to known what treatment to adopt, or if we find the patient growing worse under the influence of remedies that we think best adapted to the case, the better plan is to rely on the *vis medicatrix naturæ.* This I have repeatedly done with much satisfaction; patients in the deepest collapse having reacted without any treatment; in one case without even ice, beef-tea, or external applications." Again he says:—"When the vomiting proved obstinate, nothing but lumps of ice and teaspoonful doses of beef-tea were allowed; but frequently even these were withdrawn, and the patient permitted to remain unmolested by any kind of treatment for twelve, fifteen, or twenty hours, with the most satisfactory results, even when there was great depression of the circulation, the skin cold and cyanosed, and the rice-water discharges continuing." Other physicians have made similar statements. Dr. Hutchinson's mortality was, however, not very light. He treated one hundred and seventy patients in his hospital, of whom ninety-seven died; eighteen were in the first stage, and all recovered; seventeen were in partial collapse, of whom one died; one hundred were in complete collapse, of whom eighty-two died; five died in the consecutive stage out of nine; and nine of other diseases, including diarrhœa, out of twenty-six. It does not seem then, do the best we can, that we have any great control over this stage of collapse.

Now a few words regarding injections into the veins in collapse; not because they have been established as useful, but because the results, however temporary, are very interesting. The plan was first proposed and put in practice by Dr. Latta, of Leith, Scotland. He used a saline fluid. He adopted for his injections this formula:—two or three drachms of common salt; two scruples of subcarbonate of soda, in six pints of water, to be injected at a temperature of 112°. He selects this temperature because, he says, if it is cooler it produces a chilly feeling in the patient; and if it is much warmer, even as warm as 115°, it stimulates the heart too much, and produces a sense of weakness and fainting, The report of his first experiment I will read you in his own words:—

"The first trial was made upon an aged female, whose case was utterly hopeless (indeed he feared she would die while he was making the preparation). Having inserted the tube in the basilic vein, anxiously I awaited the effects; ounce after ounce was injected, but no visible change was produced. Still persevering, I thought she began to breathe a little less laboriously. Soon the shrunken features, and sunken eye, and fallen jaw, and face pale and cold, bearing the manifest impress of death's signet, began to glow with returning animation; the pulse, which had long ceased, returned to the wrist, at first small and quick, but by degrees becoming more and more distinct, fuller, slower, firmer; and in the short space of half an hour, when six pints had been injected, she expressed herself in a firm voice, saying she was free from all uneasiness; she actually became jocular, and fancied all she wanted was a little sleep. The extremities were warm, and all the features bore the aspect of comfort and returning health (but hear the sequel); the purging and vomiting soon returned, and she died five and a-half hours after the operation."

This unfortunately, is the history of very much the larger number upon which this experiment has been performed; of almost all. Dr. Baly says he has attempted this medication in six cases, and all have died. Dr. Streeter says that the experience of the epidemic of 1849, in England, has settled the question, has determined the inexpediency of a resort to these injections. Dr. Latta, however, reports another case. It is his first, perhaps his only, successful one. The patient was a female, aged fifty, whose condition was hardly more hopeful than that of his first case. He injected one hundred and twenty ounces of the solution, the effect of which was magical. He says:—"The restoration appeared to be complete, but the diarrhœa returned, and in three hours she was again prostrated. Again he injected one hundred and twenty ounces of the same solution, and again he had favorable results. In twelve hours three hundred and thirty ounces had been injected, and the reaction was fairly established. In forty-eight hours she smoked her pipe. She was afterwards taken to the hospital, where she had some typhoid symptoms, but finally became convalescent." Dr. Lowery states that he has performed this operation twenty-six times, and had four successful cases, and believes that the injections must be introduced slowly. If as good a result as that can be obtained with any degree of uniformity, it would seem proper to resort to the operation, because cases in extreme collapse are the only ones on which it is ever attempted—cases regarding which there can be entertained no reasonable hope from any other method of treatment.

Dr. Johnson states in his "Notes," that Dr. Mackintosh has performed this operation on 156 patients, having had 25 recoveries and 131 deaths, and thinks that such a mortality is "frightful." It corresponds very nearly with that of Dr. Lowery's cases; and supposing all the patients to have been *in extremis*, I should be unwilling to adopt this epithet. It may be true "that if all these 156 patients had been left without treatment a larger proportion would have recovered; but it is at least a fact worth knowing, that about one in six of those whose veins have been filled with this very crude imitation of blood constituents could survive the experiment. To my mind it gives the hope that a better method may be attended by greater success.

It is reported, though I don't know who performed the operation, that in 1832 there was a single successful case in this city. Drs. Gull and Baly, in summing up the history of this treatment, seem to be of the opinion that it is worthy of further trial; though Dr. Streeter regards it as completely condemned. Dr. Hutchinson used saline injections in five cases, "but with only temporary benefit." Yet he thinks that when a proper liquid is found, the proper temperature determined, the rate of injection and the quantity fixed, and when skill has overcome the difficulties attending so delicate a procedure, it may still be capable of saving lives that would otherwise be lost. He used a solution containing three drachms of common salt and one drachm of alcohol to a pint of water, at a temperature of 100° to 115° injecting two pints; and repeating when the algid symptoms returned. He also used the solution by Dr. Gull which I will give you directly. But here let me remark that it does not seem to me that a complex formula for the preparation of the fluid for injections is at all necessary. The object, of course, is to restore that which has been lost—and that is chiefly water; the evacuations from the bowels and the vomit are composed chiefly, as you will remember, of water, salt, and albumen, or albuminose. Then there is the intestinal epithelium, and nothing else of importance. In the examination of the blood, most analysts, except Dr. O'Shannessy, have reported an increased proportion of saline materials in the fluid which remains in the body. If there is then an increased proportion of saline materials, what the blood really wants is water, and not salts and water. If I were about to attempt an experiment of this sort, I would use either simple distilled water, or water with a very little common salt added.* Dr. Gull's formula is as follows—chloride of sodium, six parts by weight; chloride of potassium, the same quantity; phosphate of soda, three parts; carbonate of soda, twenty parts; of this mixture of salts one hundred and forty grains are to be dissolved in forty ounces of distilled water, and filtered. Filtering is important, because dust gets into all these substances when exposed.

I have here a pamphlet, published in Canada in 1854, containing a report of seven experiments of injecting cow's milk into the veins of persons in a collapsed state. The report is drawn up by Dr Borell; four of the trials were made by himself. He had two recoveries. A friend, who attempted it in three other cases, failed in each. The experiment was conducted in this way. A cow was brought near to the sick person, and the milk was used of the natural temperature and immediately; the bowl which held the milk was warmed and the syringe was warmed. The amount of milk used was eight to twelve ounces. My impression is, that the quantity of fluid should be considerably increased; it does not correspond at all, in these experiments, with the quantity of fluid usually lost in the dejections; yet the success is two in seven. In the other cases there was some revival of the vital powers, but they sank again; whether they would have been again revived had the injection been repeated, we cannot now decide. The pamphlet is an interesting one, containing the reasonings by which Dr. Borell came to the conclusion that it would be safe to inject milk into the veins.

Dr. A. N. Gunn, late Health Officer of this port, stated in the Academy of Medicine, since these lectures were delivered, that in 1832 injections of saline fluid were unsuccessful in the Hospital to which he was attached as assistant, and had been forbidden; and that afterwards, as he believed, three or four patients were saved by injecting into the veins pure warm water.

I return once more to Dr. Johnson, to state to you his views of the treatment of cholera. You will recollect that he regards the evacuations as salutary in eliminating the poison that oppresses and may overwhelm the vital powers. I may here say that this is not a new opinion. Dr. Johnson does not claim that it is original with him. Many physicians have been led to the same conclusion, some of whom he quotes. Dr. Hutchinson, whose paper I have often referred to, a close observer has come to the same conclusion. He says:—"Whenever a patient was admitted into the Brooklyn Cholera Hospital with copious and frequent vomiting and purging, especially of rice-water fluid, if the discharges were not involuntary, a favorable termination usually occurred, and *vice versa;* and instead of death being the consequence of such symptoms, the conclusion seems more rational that they are curative means adopted by nature to eliminate the poison from the system. Instead, therefore, of arresting the discharges, it was deemed best *to let them stop.*" Dr. Johnson goes beyond this; he is not satisfied to let them stop, but would augment them by mild cathartics, emetics, and enemata. He asks us to admit that calomel, if it cures, acts not as an alterative, or sedative, or cholagogue, but as a cathartic; that the saline mixture of Dr. Stevens is really efficacious, not because it restores the lost salts, but because it is a laxative; and he urges that the English physicians in India treated the disease with more success than the European physicians, because they were not afraid to give cathartics and at the same time call them by their right name. Dr. Johnson, in 1855, reported fifty-four cases of cholera and choleraic diarrhœa treated on this plan.—(*Epidemic Diarrhœa and Cholera, &c.*)

I have examined with some care these cases, and I am compelled to admit that I do not know any detailed reports that give better results. It is not to be forgotten, however, that the cases all occurred between the 11th of August and 28th of September, 1854; in other words, in the decline of the epidemic in London. The seventeen cases of choleraic diarrhœa all recovered; and of thirty-seven cases of cholera, all reported to have been in collapse more or less severe, twenty-three recovered. Many of these cases appear to me to have been mild; some that were considered as in collapse present very few of the symptoms of that state, but the cases numbered 2, 7, 8, and 48 were severe enough to test the virtues of any treatment, and they recovered. The great feature of the treatment is the administration of castor-oil in half-ounce doses every half-hour for several hours, then the same dose every hour, and later every two or three hours. Thus varied in frequency, these doses are continued usually from one to four days. In one instance recovery followed ounce-doses given every hour till five were taken. In one case (8) of extreme collapse, a girl nine years old took fourteen doses of an ounce each between 7 A.M. and 5½ P.M., then after an interval of nine hours she took seven half-ounce doses in ten hours; at the end of twenty-four hours half an ounce, and three days later another half-ounce, which last portion seemed to stop a diarrhœa that had continued during convalescence. She took in all eighteen and a-half ounces of castor-oil. In another case (17) of collapse, the patient took in half-ounce doses twenty-two ounces of the oil during the first day, but vomited nearly all of it; the second day and following night he took seventeen ounces, very little of which was vomited. The purging produced, the author thinks, was hazardous, but the patient revived. One patient (48) took thirty-five ounces of oil in a little more than four days, having vomited during this time 125 times. This quantity of oil, the author thinks, was twice as much as was required. The patient recovered.

I do not discover in the examination of these cases any precise rule relating to the quantity of oil to be given. If it caused vomiting it was still acting as an evacuant, and was not discontined for that reason. If it did not act in this manner, an emetic was administered in some of the cases. In full collapse Dr. Johnson seems to prefer mustard and salt as an emetic, and gives it before the oil is taken. When the oil does not prove cathartic, he uses salt and water enemata. In some instances he gave calomel with the oil, two to five grains, and if in the smaller doses, it was repeated three or four times. He applies warmth to the stomach and extremities, and uses other adjuvants, but does not speak favorably of ice and cold water, because he thinks they suspend the elimination of the poison. Such is the plan for which Dr. Johnson asks free but unprejudiced criticism. In the present state of our knowledge theoretical criticism goes for very little. The single question is, will this method save more lives than any other? In Dr. Johnson's hands it has done as well at least as any other of which I have seen the detailed reports; but the trial alone can teach us what it can do at the commencement of an epidemic. He does not claim that he has found a specific for cholera. He says, indeed in "Notes on Cholera," p. 87:—"There is no remedy which has the slightest pretensions to be considered a cure for cholera; no drug or agent which, so far as we know, will neutralize the poison or lessen its virulence. I have not the faintest hope or expectation that a specific remedy for such a disease as cholera will ever be discovered." While I would not discourage the attempts to cure cholera by evacuants, and do not doubt that Dr. Johnson's results are as satisfactory as almost any that have been published, allowance, however, being made for the stage of the epidemic to which his practice was applied, yet there is a pertinent inquiry regarding the first stage of the disease. How does it happen that agents which restrain the discharges are so efficacious in that stage? It is the almost universal practice to give them, and this of itself is one of the strongest proofs that they are proper and useful. If the poison can be eliminated by emetics and cathartics, it would seem that this is the stage in which they should be most demanded, and in which the agents which suppress discharges would be most harmful. We know but little about the modes in which animal poisons are eliminated; we do not know, indeed, that they are eliminated at all through the evacuations; we know that some of them, or rather portions of them, escape from the body, as in small-pox; but this is not an elimination that purifies the sick man, or relieves him of a single bad symptom. The poisonous principles of typhus and typhoid fevers, of measles and scarlet fever, are not eliminated before the patient begins to recover; for these diseases are communicable during convalescence. The diarrhœa of typhoid fever does not diminish the danger of the patient. Intermittent fever is suspended while the miasmatic poison still remains in the system. There is no evidence whatever that it is in the power of drugs to extract a miasmatic or an animal poison from the body, and so purify it, say what we may of depuration. Still, Dr. Johnson is not to lose the credit of his experiment. Many of his patients recovered while taking castor-oil. That fact will stand, whatever its explanation may be.

